# Supplementary material for: The relative importance of herbicide use for conservation tillage adoption by U.S. corn and soybean producers
Source: PLoS One. 2024 Nov 18;19(11):e0311960. doi: 10.1371/journal.pone.0311960 (PMC11573181; doi:10.1371/journal.pone.0311960)
Supplement: S1 Table — (DOCX) [file pone.0311960.s001.docx]

**S1 Table. Average Usage of Glyphosate and Atrazine among U.S. Field Corn (2016 and 2021) Producers Practicing Conventional Versus Conservation Tillage.**

| **Indicator** | **Tillage type** | **Glyphosate** | | | |  | **Atrazine** | | | |
| --- | --- | --- | --- | --- | --- | --- | --- | --- | --- | --- |
|  |  | **2016**  **(n=1,995 fields)** | | **2021**  **(n=968 fields)** | |  | **2016**  **(n=1,995 fields)** | | **2021**  **(n=968 fields)** | |
| Percent Crop Treated (%) | Conventional | 71% |  | 75% |  |  | 56% |  | 58% |  |
|  | Conservation | 81% |  | 76% |  |  | 63% |  | 69% |  |
|  | Difference | **+10** | *** | +1 |  |  | **+7** | ** | +11 |  |
|  |  |  |  |  |  |  |  |  |  |  |
| Annual Pounds Active Ingredient per Acre (lbs ai/acre) | Conventional | 0.93 |  | 0.82 |  |  | 0.89 |  | 0.82 |  |
|  | Conservation | 0.90 |  | 0.95 |  |  | 0.90 |  | 0.85 |  |
|  | Difference | -0.03 |  | **+0.13** | ** |  | +0.02 |  | +0.04 |  |
|  |  |  |  |  |  |  |  |  |  |  |
| Annual No. of Applications per Acre | Conventional | 0.83 |  | 0.89 |  |  | 0.65 |  | 0.73 |  |
|  | Conservation | 1.15 |  | 1.03 |  |  | 0.79 |  | 0.89 |  |
|  | Difference | **+0.32** | *** | +0.14 |  |  | **+0.14** | *** | **+0.16** | ** |
| **Pre-emergence applications** | | | | | | | | | | |
| Percent Crop Treated (%) | Conventional | 8% |  | 9% |  |  | 15% |  | 11% |  |
|  | Conservation | 29% |  | 32% |  |  | 25% |  | 28% |  |
|  | Difference | **+21** | *** | **+22** | *** |  | **+10** | *** | **+17** | *** |
|  |  |  |  |  |  |  |  |  |  |  |
| Annual Pounds Active Ingredient per Acre (lbs ai/acre) | Conventional | 0.93 |  | 0.93 |  |  | 1.00 |  | 0.84 |  |
|  | Conservation | 0.89 |  | 1.00 |  |  | 0.94 |  | 0.88 |  |
|  | Difference | -0.04 |  | +0.07 |  |  | -0.06 |  | +0.04 |  |
|  |  |  |  |  |  |  |  |  |  |  |
| Annual No. of Applications per Acre | Conventional | 0.10 |  | 0.10 |  |  | 0.16 |  | 0.12 |  |
|  | Conservation | 0.34 |  | 0.35 |  |  | 0.29 |  | 0.31 |  |
|  | Difference | **+0.24** | *** | **+0.25** | *** |  | **+0.13** | *** | **+0.19** | *** |
| **Post-emergence applications** | | | | | | | | | | |
| Percent Crop Treated (%) | Conventional | 65% |  | 69% |  |  | 43% |  | 49% |  |
|  | Conservation | 69% |  | 58% |  |  | 44% |  | 52% |  |
|  | Difference | +5 |  | **-11** | *** |  | +1 |  | +2 |  |
|  |  |  |  |  |  |  |  |  |  |  |
| Annual Pounds Active Ingredient per Acre (lbs ai/acre) | Conventional | 0.93 |  | 0.81 |  |  | 0.85 |  | 0.81 |  |
|  | Conservation | 0.91 |  | 0.92 |  |  | 0.88 |  | 0.83 |  |
|  | Difference | -0.02 |  | +0.11 |  |  | +0.03 |  | +0.02 |  |
|  |  |  |  |  |  |  |  |  |  |  |
| Annual No. of Applications per Acre | Conventional | 0.73 |  | 0.79 |  |  | 0.49 |  | 0.61 |  |
|  | Conservation | 0.82 |  | 0.66 |  |  | 0.50 |  | 0.58 |  |
|  | Difference | **+0.09** | ** | -0.13 |  |  | +0.02 |  | -0.03 |  |
