# Supplementary material for: The relative importance of herbicide use for conservation tillage adoption by U.S. corn and soybean producers
Source: PLoS One. 2024 Nov 18;19(11):e0311960. doi: 10.1371/journal.pone.0311960 (PMC11573181; doi:10.1371/journal.pone.0311960)
Supplement: S2 Table — (DOCX) [file pone.0311960.s002.docx]

**S2 Table. Average Usage of Glyphosate and 2,4-D among U.S. Soybean (2012 and 2018) Producers Practicing Conventional Versus Conservation Tillage.**

| **Indicator** | **Tillage type** | **Glyphosate** | | | | |  | **2,4-D** | | | |
| --- | --- | --- | --- | --- | --- | --- | --- | --- | --- | --- | --- |
|  |  | **2012**  **(n=1,790)** | | **2018**  **(n=2,260)** | | |  | **2012**  **(n=1,790)** | | **2018**  **(n=2,260)** | |
| Percent Crop Treated (%) | Conventional | 95% |  | | 76% |  |  | 4% |  | 7% |  |
|  | Conservation | 95% |  | | 83% |  |  | 19% |  | 23% |  |
|  | Difference | 0 |  | | **+7** | ** |  | **+15** | *** | **+16** | *** |
|  |  |  |  | |  |  |  |  |  |  |  |
| Annual Pounds Active Ingredient per Acre (lbs ai/acre) | Conventional | 0.96 |  | | 0.97 |  |  | 0.59 |  | 0.48 |  |
|  | Conservation | 0.94 |  | | 0.92 |  |  | N/D |  | 0.51 |  |
|  | Difference | -0.02 |  | | -0.05 |  |  |  |  | **+0.03** | NS |
|  |  |  |  | |  |  |  |  |  |  |  |
| Annual No. of Applications per Acre | Conventional | 1.50 |  | | 1.07 |  |  | 0.03 |  | 0.08 |  |
|  | Conservation | 1.66 |  | | 1.35 |  |  | 0.20 |  | 0.25 |  |
|  | Difference | **+0.16** | *** | | **+0.28** | *** |  | **+0.17** | *** | **+0.17** | *** |
| **Pre-emergence applications** | | | | | | | | | | | |
| Percent Crop Treated (%) | Conventional | 16% |  | | 18% |  |  | 4% |  | 6% |  |
|  | Conservation | 45% |  | | 47% |  |  | 17% |  | 21% |  |
|  | Difference | **+29** | *** | | **+29** | *** |  | **+13** | *** | **+15** | *** |
|  |  |  |  | |  |  |  |  |  |  |  |
| Annual Pounds Active Ingredient per Acre (lbs ai/acre) | Conventional | 0.96 |  | | 0.83 |  |  | 0.61 |  | 0.53 |  |
|  | Conservation | 0.87 |  | | 0.90 |  |  | 0.52 |  | 0.52 |  |
|  | Difference | **-0.09** | * | | +0.07 |  |  | -0.09 |  | -0.01 |  |
|  |  |  |  | |  |  |  |  |  |  |  |
| Annual No. of Applications per Acre | Conventional | 0.17 |  | | 0.20 |  |  | 0.03 |  | 0.06 |  |
|  | Conservation | 0.51 |  | | 0.53 |  |  | 0.18 |  | 0.23 |  |
|  | Difference | **+0.34** | *** | | **+0.32** | *** |  | **+0.15** | *** | **+0.17** | *** |
| **Post-emergence applications** | | | | | | | | | | | |
| Percent Crop Treated (%) | Conventional | 89% |  | | 66% |  |  | 1% |  | 1% |  |
|  | Conservation | 84% |  | | 65% |  |  | 2% |  | 2% |  |
|  | Difference | **-5** | ** | | -1 |  |  | **+1** | * | 0 |  |
|  |  |  |  | |  |  |  |  |  |  |  |
| Annual Pounds Active Ingredient per Acre (lbs ai/acre) | Conventional | 0.96 |  | | 0.99 |  |  | 0.49 |  | 0.26 |  |
|  | Conservation | 0.95 |  | | 0.93 |  |  | 0.45 |  | 0.41 |  |
|  | Difference | **-0.01** |  | | -0.06 |  |  | -0.04 |  | +0.14 |  |
|  |  |  |  | |  |  |  |  |  |  |  |
| Annual No. of Applications per Acre | Conventional | 1.31 |  | | 0.85 |  |  | 0.00 |  | 0.01 |  |
|  | Conservation | 1.12 |  | | 0.82 |  |  | 0.02 |  | 0.02 |  |
|  | Difference | **-0.19** | *** | | -0.03 |  |  | **+0.01** | ** | +0.01 |  |
