# Supplementary material for: The relative importance of herbicide use for conservation tillage adoption by U.S. corn and soybean producers
Source: PLoS One. 2024 Nov 18;19(11):e0311960. doi: 10.1371/journal.pone.0311960 (PMC11573181; doi:10.1371/journal.pone.0311960)
Supplement: S1 Appendix — (DOCX) [file pone.0311960.s003.docx]

**S1 Appendix. Variables Included in the CART model from the ARMS Phase 2 Surveys.**

| **Variables included in the CART model for field corn from the 2016 ARMS phase 2 survey** | | |
| --- | --- | --- |
| **Variable code in ARMS questionnaire** | **Short name** | **Description** |
| 50 | Cropped acres of corn | Total cropped acres of corn on the farm (acres) |
| 1301 | Field size | Field size (acres) |
| 702 | Conservation plan | Conservation plan specifying practice to reduce soil erosion |
| 703 | Nutrient plan | Nutrient management plan specifying practices for fertilizer/manure application |
| 704 | IPM | Pest management plan to implement IPM to control weeds, insects or disease |
| 705 | Irrigation plan | Irrigation water management plan specifying irrigation practices |
| 708 | Structural practices | Structural practices to conserve soil? (include grass waterways, terraces, grade stabilization, contour, buffer strips, etc.) |
| 709 | Nitrogen practices | Nitrogen application practices? (include split applications (at least 50% after crop emergence), applying no more than 30 days prior to planting, precision application, or using controlled release). |
| 2236 | EQIP | Is this field in an existing conservation program contract through EQIP, which you or your landlord have/expect to receive cost sharing payments, stewardship payments or incentive payments? |
| 2240 | CSP | Is this field in an existing conservation program contract through CSP, which you or your landlord have/expect to receive cost sharing payments, stewardship payments or incentive payments? |
| 2248 | Other conservation programs | Other Federal, State, Local or non-government sources |
| 2400 | Field slope | What is the slope of the field? |
| 2401 | Field soil type | What is the primary soil type |
| 1404 | HEL | Has the Natural Resource Conservation Service (NRCS) classified any part of this field as Highly Erodible? NRCS definition here: <https://www.nrcs.usda.gov/resources/guides-and-instructions/highly-erodible-land-determinations> |
| 1405 | Wetland | Have you been notified by NRCS that this field contains a wetland? |
| 2407 | Water erosion | Did you receive assistance/resources for water driven erosion? |
| 2408 | Wind erosion | Did you receive assistance/resources for wind driven erosion? |
| 2409 | Compaction | Did you receive assistance/resources for soil compaction? |
| 2410 | Poor drainage | Did you receive assistance/resources for poor soil drainage? |
| 2411 | Low organic matter | Did you receive assistance/resources for low organic matter? |
| 2412 | Water quality | Did you receive assistance/resources for water quality concerns? |
| 2413 | Other resource concerns | Did you receive assistance/resources for other resource concerns? |
| 1343/1369 | Fallow | Did you fallow in any season in the prior year? Based on code 320 for item 30 |
| 1343/1369 | Rotate | Did you rotate in any season in the prior year? Based on codes 5/6 for item 30 |
| 242 | Lime | Is lime every applied to this field? |
| 202 | Commercial nutrients | Commercial nutrients or fertilizers applied to this field for the 2016 corn crop (include those from operators, landlords, and contractors) |
| 203 | Commercial nutrient applications | Number of commercial nutrients or fertilizers applied to the field for the 2016 crop. |
| 61 | Glyphosate | Reported applying any herbicides containing Glyphosate as an active ingredient. |
| 61 | Atrazine | Reported applying any herbicides containing Atrazine as an active ingredient. |
| 1347 | Yield | Yield in bushels/acre. |
| 216 | Yield goal | What was your yield goal at planting for this field (different units, used p217 for conversion) |
| 1343/1487 | Cover crop | Was the crop planted in the fall of 2017 a cover crop? |
| 77/1301/52 | Glyphosate treated acres | Glyphosate pounds per treated acre |
| 77/1301/53 | Glyphosate treated acres post | Glyphosate pounds per treated acre after planting |
| 77/1301/54 | Glyphosate treated acres pre | Glyphosate pounds per treated acre before planting |
| 77/1301/55 | Glyphosate acre treatments | Total treatments of Glyphosate per field acre |
| 77/1301/56 | Glyphosate acre treatments post | Total treatments of Glyphosate per field acre after planting |
| 77/1301/57 | Glyphosate acre treatments pre | Total treatments of Glyphosate per field acre before or at planting |
| 77/1301/58 | Atrazine treated acres | Atrazine pounds per treated acre |
| 77/1301/59 | Atrazine treated acres post | Atrazine pounds per treated acre after planting |
| 77/1301/60 | Atrazine treated acres pre | Atrazine pounds per treated acre before planting |
| 77/1301/61 | Atrazine acre treatments | Total treatments of Atrazine per field acre |
| 77/1301/62 | Atrazine acre treatments post | Total treatments of Atrazine per field acre after planting |
| 77/1301/63 | Atrazine acre treatments pre | Total treatments of Atrazine per field acre before or at planting |
| 77/1301/64 | Glyphosate % base | Percent of base acres treated with Glyphosate |
| 77/1301/65 | Glyphosate % base post | Percent of base acres treated with Glyphosate after planting |
| 77/1301/66 | Glyphosate % base pre | Percent of base acres treated with Glyphosate before planting |
| 77/1301/67 | Atrazine % base | Percent of base acres treated with Atrazine |
| 77/1301/68 | Atrazine % base post | Percent of base acres treated with Atrazine after planting |
| 77/1301/69 | Atrazine % base pre | Percent of base acres treated with Atrazine before planting |
| 805 | Herbicides post emergence | Herbicides were applied to this field after weeds emerged? |
| 803 | Herbicides pre-emergence | Herbicides were applied to this field before weeds emerged? |
| 841 | Lab for pest or plant identification | Did you use the services of a diagnostic laboratory for pest identification or soil plant tissue pest analysis for this field? |
| 844 | Rotate crops | Did you rotate crops in the field during the past three years? |
| 845 | Ground cover | Did you maintain ground cover, mulches, or other physical barriers |
| 846 | Crop variety | Did you choose a crop variety because of specific resistance to a certain pest? |
| 848 | Cross infestation | Did you plan planting locations to avoid cross infestation of pests? |
| 849 | Adjust dates | Did you adjust planting or harvesting dates to manage or reduce the spread of pests in the field? |
| 850 | Fence line maintenance | Did you chop/spray/mow/plow/burn field edges/lanes/ditches/roadways/fence lines to manage or reduce the spread of pests in the field? |
| 851 | Clean equipment | Did you clean equipment for the specific purpose of managing or reducing the spread of pests in fields? |
| 852 | Rows and planting density | Did you adjust row spacing, plant density or row directions for the specific purpose of managing or reducing the spread of pests in fields? |
| 854 | Treated seeds | Did you treat seeds for insects or disease control after you purchased the seed for this field for the specific purpose of managing or reducing the spread of pests? |
| 855 | Beneficial habitat | Did you maintain beneficial insect or vertebrate habitat for the specific purpose of managing or reducing the spread of pests? |
| 857 | Flamer | Did you use a flamer to kill weeds for the specific purpose of managing or reducing the spread of pests? |
| 856 | Buffer (harvest) from non-organics | Did you maintain buffer strips or border rows to isolate corn from non-organic crops or land, or did you take a buffer harvest for the specific purpose of managing or reducing the spread of pests? |
| 865 | Plant earlier/later | Did you plant earlier or later to avoid weeds? |
| 834 | Decline Glyphosate | Have you noticed a decline in the effectiveness of glyphosate (e.g. Roundup) in controlling weeds in this field? |
| 835 | Decline Glyphosate (year) | What was the first year you noticed a decline in the effectiveness of Glyphosate |
| 837 | Stop planting Glyphosate | After noticing the decline in effectiveness of Glyphosate in controlling weeds on this field, did you stop planting Glyphosate? |
| 839 | Change tillage practices Glyphosate | After noticing the decline in effectiveness of Glyphosate in controlling weeds on this field, did you change tillage practices? |
| 1840 | Increase use Glyphosate | After noticing the decline in effectiveness of Glyphosate in controlling weeds on this field, did you increase Glyphosate use? |
| 1885 | Increase use Atrazine | After noticing the decline in effectiveness of Glyphosate in controlling weeds on this field, did you increase Atrazine use? |
| 1847 | Decrease use Glyphosate | After noticing the decline in effectiveness of Glyphosate in controlling weeds on this field, did you decrease Glyphosate use? |
| 1887 | Decrease use Atrazine | After noticing the decline in effectiveness of Glyphosate in controlling weeds on this field, did you decrease Atrazine use? |
| 1854 | Discontinue Glyphosate | After noticing the decline in effectiveness of Glyphosate in controlling weeds on this field, did you discontinue Glyphosate use? |
| 1879 | Discontinue Atrazine | After noticing the decline in effectiveness of Glyphosate in controlling weeds on this field, did you discontinue Atrazine use? |
| 1861 | Did not change Glyphosate | After noticing the decline in effectiveness of Glyphosate in controlling weeds on this field, did you not change Glyphosate use? |
| 1881 | Did not change Atrazine | After noticing the decline in effectiveness of Glyphosate in controlling weeds on this field, did you not change Atrazine use? |
| 1868 | Did not use Glyphosate | After noticing the decline in effectiveness of Glyphosate in controlling weeds on this field, did you not use Glyphosate at all? |
| 1883 | Did not use Atrazine | After noticing the decline in effectiveness of Glyphosate in controlling weeds on this field, did you not use Atrazine at all? |
| 888 | Move equipment (resistance) | Considering each year you planted a Glyphosate resistant crop on this field, have you ever cleaned equipment between moving from one field to the next to reduce the rate that Glyphosate resistance develops in weeds on this field? |
| 892 | Rotate crops (resistance) | Considering each year you planted a Glyphosate resistant crop on this field, have you ever rotated crops from one field to the next to reduce the rate that Glyphosate resistance develops in weeds on this field? |
| 864 | Fallow to manage insects | Was this field left in fallow in 2015 to help manage insects on this field? |
| 886 | Control weeds early | Considering each year you planted a glyphosate resistant crop on this field, have you ever tried to control weeds early in the season to prevent resistance to Glyphosate? |
| 887 | Control weed escapes | Considering each year you planted a glyphosate resistant crop on this field, have you ever tried to control weed escapes to prevent resistance to Glyphosate? |
| 889 | Use non-Glyphosate herbicide | Considering each year you planted a glyphosate resistant crop on this field, have you used herbicides other than Glyphosate to prevent resistance to Glyphosate? |
| 891 | Use label application | Considering each year you planted a glyphosate resistant crop on this field, have you used the herbicide label recommended application rate in order to prevent resistance to Glyphosate? |
| 827 | Did pests cause yield loss | Did any pests (weeds, insects, pathogens, animals) cause any yield loss on this field in spit of your pest control efforts? |
| State* | U.S. state | 1 of 50 U.S. states. |
| ERSREGN* | ERS resource region | 1. Heartland, 2. Northern Crescent, 3. Great Plains, 4. Prairie Gateway, 5. Eastern Uplands, 6. Southern Seaboard, 7. Fruitful Rim, 8. Basin and Range, 9. Mississippi Portal |

| **Variables included in the CART model for soybeans from the 2018 ARMS phase 2 survey** | | |
| --- | --- | --- |
| **Variable code in ARMS questionnaire** | **Short name** | **Description** |
| 50 | Cropped acres | Total cropped acres of soybeans on the farm (acres) |
| 1343/1369 | Rotate | Did you rotate in any season in the prior year? Based on codes 5/6 for item 30 |
| 2236 | EQIP | Is this field in an existing conservation program contract through EQIP, which you or your landlord have/expect to receive cost sharing payments, stewardship payments or incentive payments? |
| 2240 | CSP | Is this field in an existing conservation program contract through CSP, which you or your landlord have/expect to receive cost sharing payments, stewardship payments or incentive payments? |
| 2400 | Field slope | What is the slope of the field? |
| 2401 | Field soil type | What is the primary soil type? |
| 1404 | HEL | Has the Natural Resource Conservation Service (NRCS) classified any part of this field as Highly Erodible? |
| 1405 | Wetland | Have you been notified by NRCS that this field contains a wetland? |
| 2407 | Water erosion | Did you receive assistance/resources for water driven erosion? |
| 2408 | Wind erosion | Did you receive assistance/resources for wind driven erosion? |
| 2409 | Compaction | Did you receive assistance/resources for soil compaction? |
| 2410 | Poor drainage | Did you receive assistance/resources for poor soil drainage? |
| 2411 | Low organic matter | Did you receive assistance/resources for low organic matter? |
| 2412 | Water quality | Did you receive assistance/resources for water quality concerns? |
| 2413 | Other resource concerns | Did you receive assistance/resources for other resource concerns? |
| 242 | Lime | Is lime every applied to this field |
| 202 | Commercial nutrients | Commercial nutrients or fertilizers applied to this field for the 2016 corn crop (include those from operators, landlords, and contractors) |
| 203 | Commercial nutrient applications | Number of commercial nutrients or fertilizers applied to the field for the 2016 crop. |
| 61 | Glyphosate | Reported applying any herbicides containing Glyphosate as an active ingredient. |
| 61 | 2-4,D | Reported applying any herbicides containing 2-4,D as an active ingredient. |
| 77/1301/52 | Glyphosate acre treatments | Total treatments of Glyphosate per field acre |
| 77/1301/53 | Glyphosate acre treatments post | Total treatments of Glyphosate per field acre after planting |
| 77/1301/54 | Glyphosate acre treatments pre | Total treatments of Glyphosate per field acre before or at planting |
| 77/1301/55 | 2,4-D acre treatments | Total treatments of 2,4-D per field acre |
| 77/1301/56 | 2,4-D acre treatments post | Total treatments of 2,4-D per field acre after planting |
| 77/1301/57 | 2,4-D acre treatments pre | Total treatments of 2,4-D per field acre before or at planting |
| 77/1301/58 | Glyphosate treated acres | Glyphosate pounds per treated acre |
| 77/1301/59 | Glyphosate treated acres post | Glyphosate pounds per treated acre after planting |
| 77/1301/60 | Glyphosate treated acres pre | Glyphosate pounds per treated acre before planting |
| 77/1301/61 | 2,4-D treated acres | 2,4-D pounds per treated acre |
| 77/1301/62 | 2,4-D treated acres pre | 2,4-D pounds per treated acre after planting |
| 77/1301/63 | 2,4-D treated acres post | 2,4-D pounds per treated acre before planting |
| 77/1301/64 | Glyphosate % base | Percent of base acres treated with Glyphosate |
| 77/1301/65 | Glyphosate % base pre | Percent of base acres treated with Glyphosate after planting |
| 77/1301/66 | Glyphosate % base post | Percent of base acres treated with Glyphosate before planting |
| 77/1301/67 | 2,4-D % base | Percent of base acres treated with 2,4-D |
| 77/1301/68 | 2,4-D % base pre | Percent of base acres treated with 2,4-D after planting |
| 77/1301/69 | 2,4-D % base post | Percent of base acres treated with 2,4-D before planting |
| 1347 | Yield | Bushels per acre |
| 1311 | Yield goal | What was your yield goal at planting for this field (different units, used conversion factors provided) |
| 1343/1470 | Cover Crop | Was the crop planted in the fall of 2015 a cover crop? |
| 805 | Herbicides post emergence | Herbicides were applied to this field after weeds emerged? |
| 803 | Herbicides pre emergence | Herbicides were applied to this field before weeds emerged? |
| 41 | Lab for pest or plant identification | Did you use the services of a diagnostic laboratory for pest identification or soil plant tissue pest analysis for this field? |
| 844 | Rotate crops (resistance) | Considering each year you planted a Glyphosate resistant crop on this field, have you ever rotated crops from one field to the next to reduce the rate that Glyphosate resistance develops in weeds on this field? |
| 845 | Ground cover | Did you maintain ground cover, mulches, or other physical barriers |
| 846 | Crop variety | Did you choose a crop variety because of specific resistence to a certain pest? |
| 848 | Cross infestation | Did you plan planting locations to avoid cross infestation of pests? |
| 849 | Adjust dates | Did you adjust planting or harvesting dates to manage or reduce the spread of pests in the field? |
| 850 | Fence line maintenance | Did you chop/spray/mow/plow/burn field edges/lanes/ditches/roadways/fence lines to manage or reduce the spread of pests in the field? |
| 851 | Clean equipment | Did you clean equipment for the specific purpose of managing or reducing the spread of pests in fields? |
| 852 | Rows and planting density | Did you adjust row spacing, plant density or row directions for the specific purpose of managing or reducing the spread of pests in fields? |
| 854 | Treated seeds | Did you treat seeds for insects or disease control after you purchased the seed for this field for the specific purpose of managing or reducing the spread of pests? |
| 855 | Beneficial habitat | Did you maintain beneficial insect or vertebrate habitat for the specific purpose of managing or reducing the spread of pests? |
| 857 | Flamer | Did you use a flamer to kill weeds for the specific purpose of managing or reducing the spread of pests? |
| 856 | Buffer (harvest) from non-organics | Did you maintain buffer strips or border rows to isolate corn from non-organic crops or land, or did you take a buffer harvest for the specific purpose of managing or reducing the spread of pests? |
| 865 | Plant earlier/later | Did you plant earlier or later to avoid weeds? |
| 2022 | Decline Glyphosate | Have you noticed a decline in the effectiveness of glyphosate (e.g. Roundup) in controlling weeds in this field? |
| 2023 | Decline Glyphosate (year) | What was the first year you noticed a decline in the effectiveness of Glyphosate |
| 2024 | Stop planting Glyphosate | After noticing the decline in effectiveness of Glyphosate in controlling weeds on this field, did you stop planting Glyphosate? |
| 2025 | Change tillage practices Glyphosate | After noticing the decline in effectiveness of Glyphosate in controlling weeds on this field, did you change tillage practices? |
| 2026 | Alternative Glyphosate | Did you switch to an alternative herbicide? |
| 1301 | Field size | Size of field (in acres) |
| 864 | Fallow to manage insects | Was this field left in fallow in 2015 to help manage insects on this field? |
| 1343/1369 | Fallow | Did you fallow in any season in the prior year? Based on code 320 for item 30 |
| State* | U.S. state | 1 of 50 U.S. states. |
| ERSREGN* | ERS resource region | 1. Heartland, 2. Northern Crescent, 3. Great Plains, 4. Prairie Gateway, 5. Eastern Uplands, 6. Southern Seaboard, 7. Fruitful Rim, 8. Basin and Range, 9. Mississippi Portal |
