# Supplementary material for: The relative importance of herbicide use for conservation tillage adoption by U.S. corn and soybean producers
Source: PLoS One. 2024 Nov 18;19(11):e0311960. doi: 10.1371/journal.pone.0311960 (PMC11573181; doi:10.1371/journal.pone.0311960)
Supplement: Attachment — Submitted filename: Response to PlosOne Reviewers_09_12_2024.pdf [file pone.0311960.s005.pdf]

September 12, 2024

Rebecca Nemec Boehm, PhD
Deputy Director, Climate and Environmental Markets
Office of Energy and Environmental Policy
United States Department of Agriculture

Dear *PlosOne* Editors,

Thank you very much for the very constructive, useful comments on our first manuscript. This document describes the responses we have made to Reviewer #1 and Reviewer #2 comments we received. We worked diligently to address all the reviewer comments and we believe the revised manuscript is significantly improved. We are looking forward to working with you in the future on publishing this manuscript in *PlosOne*.

Sincerely,

Rebecca Nemec Boehm, PhD

RESPONSE TO REVIEWER COMMENTS

Reviewer #1

Comment: The manuscript prepared in good manner. However, it requires some corrections to improve the quality. For instance, it is very important to provide brief information about weed problems at the beginning of the introduction section. For other suggestions, see the comments and suggestions in the attached MS file

Response: *Thank you for your review and suggestions for improving the manuscript we submitted. As per the reviewer's suggestion, we have added a new paragraph to the Introduction that provides more general information about the problems caused by weeds and how herbicides have provided a solution in agricultural production. In addition, we have carefully incorporated references that provide foundational information about weeds and are relevant to the crops (corn and soybeans) discussed in our manuscript, aligning more closely with the focus of our study. We have responded to the remainder of your comments directly in the MS file.*

Should the reviewer require additional revisions to this text, please inform us. Thank you.

Reviewer #2

Comment #1: The abstract needs to be rewritten, and the proper motivation followed by clear objectives and major findings with quantitative support need to be provided in the revised abstract.

> ***Response:*** *The abstract has been significantly rewritten to address this comment. We have included the proper motivation and clear objectives, as suggested. We have also added brief mentions of the quantitative results from the pairwise mean comparison tests and the CART analysis, while keeping the content concise due to space limitations.*

Comment #2: The introduction needs to be revised, and the research gap is not clear. Hence, authors are advised to rewrite/ update the introduction to clearly present the research gap, and in continuing with that, proper objectives must be formulated.

> ***Response:*** *We have significantly rewritten the Introduction to make the research gap and the objectives clearer and given Comment #1 and our response, we made sure that the Abstract and the Introduction text are aligned. In particular we now more clearly emphasize in both the Introduction and Abstract that the goal of the paper is to develop a more recent and comprehensive understanding of how herbicide usage levels predict conservation tillage use. We also highlight that the CART analysis is a novel method that has not been employed in this research area and it offers distinct advantages over traditional statistical methods or those of regression analysis, which is why it is a contribution to the literature on this topic.*

Comment #3: Authors must avoid using the first person in the technical writing process (avoid the use of i/we//our/us, etc.)

> ***Response:*** *We have revised the manuscript so it is no longer written in the first person.*

Comment #4: All the headings and subheadings must be properly numbered starting from the introduction with no 1.

> ***Response:*** *All headings and subheadings are now properly numbered starting with the Introduction as number 1.*

Comment #5: The explanation with the figures caption can be kept as text in the main body of the manuscript and can be discussed properly.

> ***Response:*** *We apologize for any confusion regarding the figure captions. We are uncertain how to address this comment, as the figure captions are already included in the main body of the manuscript, in accordance with our interpretation of the preparation instructions. Can you clarify what specific adjustments need to be made to the figure captions? Thank you.*

Comment #6: The results and analysis must be presented and discussed in appropriate sections. Here, the authors have presented and analyzed the results in the prior sections. Hence, it is suggested that the authors follow the IMRAD paper structure for better presentation and understanding.

> ***Response:*** *Apologies for the confusion here. We believe the primary cause of the confusion is the description of subheadings in the methods section as well as the lack of heading and subheading numbers throughout the manuscript, especially in the results and discussion sections. To address this, we have revised the subheadings in the methods, results, and discussion sections to clarify that those sections only outline the methodology we used to evaluate the importance of herbicide usage to conservation tillage. We have also added a short section in the methods to describe how we evaluated the prevalence and trends in conservation tillage and herbicide usage, ensuring better alignment with the corresponding results section.*

Comment #7: The analysis of the obtained results is very poor, and there is no comparison with any existing/ published work. Hence, the authors are suggested to update the results, compare them in the discussion section, and provide appropriate reasoning for the improved behavior in the proposed method.

> ***Response:*** Thank you for your constructive comments. We have substantially revised the discussion section and added further comparisons to prior studies where applicable. We have also more clearly articulated the improved performance of the CART analysis. We believe these revisions have significantly enhanced the manuscript.

Comment #8: Conclusions must be rewritten (in max 200 words), and they must convey the major findings of the research with quantitative support.

> ***Response:*** *We have revised the Conclusion to meet the 200-word limit. We have added quantitative information into the text, as well as references to key figures and tables that provide quantitative support in cases where we could not briefly summarize all quantitative results in the text due to the word count constraint. Apologies that it was over the word limit in the previous draft.*
