# Supplementary material for: The relative importance of herbicide use for conservation tillage adoption by U.S. corn and soybean producers
Source: PLoS One. 2024 Nov 18;19(11):e0311960. doi: 10.1371/journal.pone.0311960 (PMC11573181; doi:10.1371/journal.pone.0311960)
Supplement: Attachment — Submitted filename: Herbicides and conservation tillage_03_06_24_Reviewer.docx [file pone.0311960.s004.docx]

**Title page**

**Short title**

Conservation Tillage Adoption and Herbicide Use

**Title**

The Relative Importance of Herbicide Use for Conservation Tillage Adoption by U.S. Corn and Soybean Producers

**Authors**

Laura Dodson, USDA Economic Research Service, Washington, DC

Fengxia Dong, USDA Economic Research Service, Washington, DC

Rebecca Nemec-Boehm, USDA Office of the Chief Economist, Washington, DC*

Cameron Douglass, USDA Office of Pest Management Policy, Washington, DC

Michelle Ranville, USDA Office of Pest Management Policy, Washington, DC

Ryan Olver, Tokyo International University, Tokyo, Japan

*Corresponding author email address: Rebecca.nemec@usda.gov

**Disclaimer**

This research was supported [in part] by the U.S. Department of Agriculture, Economic Research Service. The findings and conclusions in this publication are those of the author(s) and should not be construed to represent any official USDA or U.S. Government determination or policy.

**Abstract**

The use of herbicides is widespread in modern agricultural production, not only to control weeds prior to and after planting, but also to “burndown” weeds in the spring as part of practicing conservation tillage (i.e., as an alternative to conventional tillage). Older studies examined the relationship between herbicide use levels and tillage practices (i.e., conservation versus conventional tillage), but findings were somewhat ambiguous and did not fully consider this relationship in the context of other economic and agronomic factors. This study examines this relationship in two ways using data from the US Department of Agriculture Agricultural Resource Management Survey (ARMS), which provides nationally representative data on the production practices for major commodities. First, the study provides the latest trends and prevalence of conservation tillage nationally, across production regions and states, and conducts statistical comparisons of different measures of herbicide usage by tillage practices for field corn and soybean. The study finds that conservation tillage has increased nationally over time for both crops and that herbicide usage, for some measures in some years, is significantly higher on acres using conservation versus conventional tillage. Second, the study uses Classification and Regression Tree (CART) analysis to examine the importance of herbicide usage as a predictor of tillage practices relative to a wide range of other economic and agronomic factors. The CART analysis indicates that pre-emergent use of glyphosate was the strongest predictor of conservation tillage adoption for field corn in 2016 and soybeans in 2018, but other factors such as use of crop rotations, region, and farm size are predictors of conservation tillage. Our results demonstrate the importance of herbicides to the adoption of conservation tillage in field corn and soybeans, providing timely information for policymakers as they consider proposals to expand conservation tillage to achieve climate or other policy objectives.

**Introduction**

Field crops suffer from biotic stress caused by invasive plants called weeds (*). In this situation, weeds detrimentally influence on crop growth and productivity (**). Weeds remove nutrients from the soil and deprive crop plants of them, thus reducing productivity and quality (***). Therefore, successful crop management practices should involve decisive tactics to combat weeds and remarkably minimize their issues (****). Great successes have been achieved in controlling weeds in cereal crops using herbicides (*****). Specifically, herbicides usage in corn exhibited distinctive efficiency for controlling different weed types, keeping the environmental growth factors available for desirable plants (******).

(*)

El-Metwally IM Geries L, Saudy HS (2022) Interactive effect of soil mulching and irrigation regime on yield, irrigation water use efficiency and weeds of trickle–irrigated onion. Archiv. Agron. Soil Sci. 68:1103-1116. <https://doi.org/10.1080/03650340.2020.1869723>

Rizk TY, kholousy ASO, Saudy HS., Sultan ShS, Abd Alwahed SHA (2023) Breaking dormancy and enhancing germination of *Avena sterilis* L. and *Amaranthus retroflexus* L. weeds by gibberellic acid and potassium nitrate to keep soil and crops healthy. Gesunde Pflanzen. 75:757-763.<https://doi.org/10.1007/s10343-022-00780-6>

Saudy HS (2014) Chlorophyll meter as a tool for forecasting wheat nitrogen requirements after application of herbicides. Archiv. Agron. Soil Sci 60:1077-1090. <https://doi.org/10.1080/03650340.2013.866226>

(**)

El-Metwally IM, Saudy HS (2021) Interactional impacts of drought and weed stresses on nutritional status of seeds and water use efficiency of peanut plants grown in arid conditions. Gesunde Pflanzen. 73:407-416. <https://doi.org/10.1007/s10343-021-00557-3>

El-Metwally IM, Saudy HS, Elewa TA (2022) Natural plant by-products and mulching materials to suppress weeds and improve sugar beet (*Beta vulgaris* L.) yield and quality. J Soil Sci Plant Nutr. 22:5217-5230 <https://doi.org/10.1007/s42729-022-00997-4>

Saudy HS (2015) Maize–cowpea intercropping as an ecological approach for nitrogen-use rationalization and weed suppression. Archiv. Agron. Soil Sci 61:1-14. <https://doi.org/10.1080/03650340.2014.920499>

(***)

Saudy HS, Mubarak M (2015) Mitigating the detrimental impacts of nitrogen deficit and fenoxaprop-p-ethyl herbicide on wheat using silicon. Comm. Soil Sci. Plant Anal 46:913-923. <https://doi.org/10.1080/00103624.2015.1011753>

Saudy HS, El-Metwally IM, Abd El-Samad GA (2020) Physio–biochemical and nutrient constituents of peanut plants under bentazone herbicide for broad–leaved weed control and water regimes in dry land areas. J. Arid Land, 12, (4): 630-639. <https://doi.org/10.1007/s40333-020-0020-y>

Saudy HS, El-Metwally IM, Shahin MG (2021) Co–application effect of herbicides and micronutrients on weeds and nutrient uptake in flooded irrigated rice: Does it have a synergistic or an antagonistic effect?. Crop Prot. 149, 105755. <https://doi.org/10.1016/j.cropro.2021.105755>

Saudy HS, El-Metwally IM, Sobieh ST, Abd-Alwahed SHA (2022) Mycorrhiza, charcoal, and rocket salad powder as eco-friendly methods for controlling broomrape weed in inter-planted faba bean with flax. J Soil Sci Plant Nutr. 22:5195-5206 <https://doi.org/10.1007/s42729-022-00995-6>

(****)

El-Bially MA, El-Metwally IM, Saudy HS, Aisa KH, Abd El-Samad GA (2023) Mycorrhiza-inoculated biochar as an eco-friendly tool improves the broomrape control efficacy in two faba bean cultivars. Rhizosphere 26:100706 <https://doi.org/10.1016/j.rhisph.2023.100706>

El-Metwally IM, Saudy HS (2021) Interactive application of zinc and herbicides affects broad–leaved weeds, nutrient uptake, and yield in rice. J Soil Sci Plant Nutr. 21:238-248. <https://doi.org/10.1007/s42729-020-00356-1>

Saudy HS, El-Metwally IM (2009) Weed management under different patterns of sunflower-soybean intercropping. J. Central Eur. Agric., 10:41-52. <https://hrcak.srce.hr/41577>

Saudy HS, Hamed MF, El–Metwally IM, Ramadan KhA, Aisa KH (2021) Assessing the effect of biochar or compost application as a spot placement on broomrape control in two cultivars of faba bean. J Soil Sci Plant Nutr. 21:1856-1866. <https://doi.org/10.1007/s42729-021-00485-1>

(*****)

Saudy HS, El-Metwally IM, Shahin MG (2021) Co–application effect of herbicides and micronutrients on weeds and nutrient uptake in flooded irrigated rice: Does it have a synergistic or an antagonistic effect?. Crop Prot. 149, 105755. <https://doi.org/10.1016/j.cropro.2021.105755>

Saudy HS, Mubarak M (2015) Mitigating the detrimental impacts of nitrogen deficit and fenoxaprop-p-ethyl herbicide on wheat using silicon. Comm. Soil Sci. Plant Anal 46:913-923. <https://doi.org/10.1080/00103624.2015.1011753>

Saudy HS, El-Metwally IM (2023) Effect of irrigation, nitrogen sources and metribuzin on performance of maize and its weeds. Comm. Soil Sci. Plant Anal. 54:22-31 <https://doi.org/10.1080/00103624.2022.2109659>

(******)

Saudy HS, El-Bially, MA, Ramadan KhA, Abo El–Nasr EKh, Abd El-Samad GA (2021a) Potentiality of soil mulch and sorghum extract to reduce the biotic stress of weeds with enhancing yield and nutrient uptake of maize crop. Gesunde Pflanzen. 73:555-564. <https://doi.org/10.1007/s10343-021-00577-z>

Saudy HS, El-Metwally IM (2023) Effect of irrigation, nitrogen sources and metribuzin on performance of maize and its weeds. Comm. Soil Sci. Plant Anal. 54:22-31 <https://doi.org/10.1080/00103624.2022.2109659>

Broad spectrum herbicides (i.e., for both grasses and broadleaved plants) such as 2,4-D, atrazine, and glyphosate are in widespread use in U.S. commodity production to control weeds, which in turn help ensure high crop yields [1–3]. These broad spectrum herbicides are also used by producers practicing conservation tillage (hereafter defined collectively as no till or mulch/reduced tillage) most typically as a “burndown” tool to clear fields of weeds and other existing plants prior to or at the time of cash crop planting [4–8]. With conventional tillage, producers plow their fields and mechanically remove all plant residue from the top layer of soil instead of applying herbicide for this purpose.

While herbicides play a role in facilitating the implementation of conservation tillage, many agronomic and economic factors are also important. For example, producers may have a desire to adopt conservation tillage to preserve soil moisture, prevent soil erosion, or reduce fuel, labor and machinery costs [9,10]. Farm soil type, topsoil depth and local climatic conditions, as well as availability and price of machinery needed to implement conservation tillage, age of existing tillage machinery and the sunk costs associated with it, and the price and availability of herbicides are additional factors [11–14]. Reduced tillage also has numerous soil health benefits including carbon sequestration and reduces agricultural carbon emissions by reducing the amount of fuel needed for pre-plant tillage operations, which may be another reason producers choose to adopt this practice [15–18].

How herbicides in particular factor into the decision to adopt conservation tillage has been a question of research and policy relevance for decades. Much of the research on this topic was motivated by the desire to inform policymakers about the difference in herbicide usage or intensity between conservation and conventional tillage when federal lawmakers authorized conservation compliance policies that required less tillage for producers operating on highly erodible land [19] and when post emergent (i.e., applied after emergence of the cash crop) herbicides and herbicide resistant crops became commercially available and more popular in U.S. crop production (Benbrook 2016; Kniss 2018). Prior research has not quantitatively compared the strength of the relationship between conservation tillage and herbicide usage in the context of other agronomic and economic factors. Past research only qualitatively suggested that other factors (e.g., weather, soil type, endemic weed issues, etc.) were likely more influential to conservation tillage adoption and implementation than herbicide use [21].

This prior research on field corn and soybean production also did not consistently find higher rates or increased intensity of herbicide use among producers using conservation tillage compared to conventional tillage. A 1998 analysis by USDA’s Economic Research Service and the Natural Resource Conservation Service using Cropping Practices Survey data collected between 1990 and 1995 among corn and soybean farmers found that herbicide application rates for field corn, soybean and winter wheat producers were higher (11-12% for corn and soybeans, and 68% higher for winter wheat) for those using conservation tillage systems versus conventional tillage [22]. However, using data collected from USDA’s Area Studies Survey of corn farmers in four watersheds in Indiana, Central Nebraska, Eastern Iowa and Southern Illinois, Fuglie (1999) found that herbicide usage was not always higher on fields with conservation tillage systems compared to those with conventional tillage. Fuglie’s results were consistent with earlier studies comparing herbicide usage across producers using conservation and conventional tillage [23–25].

More recently, data from the USDA Agricultural Resources Management Survey (ARMS) and the Conservation Technology Information Center (covering Illinois, Indiana, Iowa, Kansas, Michigan, Minnesota, Missouri, Nebraska, North Dakota, Ohio, South Dakota, and Wisconsin) were combined to evaluate the relationship between adoption of herbicide tolerant soybeans, conservation tillage, and “quality-adjusted herbicide use”. This study found that adoption of the herbicide resistant crop induced farmers to adopt conservation tillage while also decreasing “quality-adjusted herbicide use,” a derived parameter the authors calculated based on annual per acre application rates, soil half-lives (a measure of the persistence of pesticides in soils), and “chronic toxicity scores” [12,26]. Shortly thereafter a study using Kynetec’s AgroTrak data from 1998 to 2011 covering all U.S. Crop Reporting Districts found significantly higher usage of herbicides for no-till producers compared to other producers [27].

Studies on the cost of herbicides for producers using conventional versus conservation tillage also provides evidence on the difference in usage of herbicides across tillage practices. A study by the Environmental Defense Fund, the National Corn Growers Association Soil Health Partnership and K-Coe Isom surveyed seven corn and soybean farmers in the Midwest in 2021, finding that herbicide costs were $6.00 per acre higher for those using conservation tillage versus conventional tillage [28]. A similar case-study approach of three corn, soybean, and wheat farmers found lower costs for chemicals, including herbicides, for those implementing conservation tillage versus conventional tillage [29]. It is important to note that these two studies were very small in scale, not representative of U.S. producers and so results have relatively limited generalizability. They are nonetheless worth mentioning given that they provide newer data on the relationship between herbicide usage and conservation tillage.

To add to this literature on this topic and to better understand the relationship between conservation tillage and herbicide usage we document recent temporal and geographic trends in 2,4-D, atrazine, and glyphosate usage and the adoption of conservation tillage (which includes both no till and mulch tillage) among U.S. field corn and soybean producers using U.S Department of Agriculture’s Agricultural Resource Management Survey (ARMS) data collected in 2012 and 2018 (for soy) and 2016 and 2021 (for field corn). We focus on field corn and soybeans for this study given their overall contribution to U.S. agricultural crop production (in terms of both value and total acres planted) and the relative abundance of USDA data on tillage practices and herbicide usage data for these crops. We focus in this paper on the usage of atrazine and glyphosate in field corn, and the usage of 2,4-D and glyphosate in soybeans, given the relatively high pre- and post-emergence usage of these herbicides in these two crops and pending regulatory changes for these herbicides under consideration by the US Environmental Protection Agency [30].

We examine the relationship between conservation tillage and herbicide usage in two ways. First, we statistically compare mean herbicide usage by tillage practice type using three different indicators for field corn (in 2016 and 2021) and soybeans (in 2012 and 2018). Second, because adoption of tillage practices and its relationship to herbicide usage is confounded by a variety of farm-level factors, we use a Classification and Regression Tree (CART) model to analyze how usage of these herbicides and other agronomic and economic factors are associated with adoption of conservation tillage by field corn and soybean producers. Through the CART analysis we identify the relative importance of herbicide usage to conservation tillage among field corn and soybean producers in the context of numerous other agronomic and economic factors.

**Materials and Methods**

**Data**

This study uses data from USDA Economic Research Service (ERS) and National Agricultural Statistics Service (NASS) Agricultural Resource Management Survey (ARMS) for field corn for 2016 and 2021 and soybeans for 2012 and 2018. ARMS is a nationally representative survey carried out in three phases that targets the minimum number of states that represent 90 percent of all U.S. field corn and soybean acreage, which allow our results to be nationally representative of U.S. field corn and soybean acreage. Phase 1 screens farms for eligibility based on the commodity they are producing in the survey year and if they expect to or normally sell >$1,000 worth of agricultural products. Phase 2 asks detailed questions about quantity of inputs used, production practices, and production costs for a randomly selected field on their farm operation. Phase 3 collects farm-level information about farm demographic characteristics and finances. The states in the 2016 and 2021 survey for field corn include: North Dakota, South Dakota, Kansas, Pennsylvania, Ohio, Indiana, Wisconsin, Kentucky, North Carolina, Illinois, Nebraska, Iowa, Michigan, Colorado, Minnesota, Missouri, Texas, and New York. States included in 2012 and 2018 for the soybean survey include: Tennessee, Ohio, Kentucky, North Carolina, Kansas, Arkansas, Nebraska, Michigan, Minnesota, Mississippi Wisconsin, Missouri, Louisiana, South Dakota, Indiana, Illinois, North Dakota, Iowa, Virginia. For this analysis we used data from Phase 2 for field corn (2016 and 2021) and soybeans (2012 and 2018).

The main outcome variable of interest is whether the producer was implementing conventional tillage, mulch tillage, or no till on their ARMS sample field during the survey year. We used the Soil Tillage Intensity Rating (STIR) to classify producers as implementing conventional tillage, mulch tillage, or no-till on their sample field. STIR, a measure developed by USDA’s Natural Resource Conservation Service (NRCS), quantifies soil disturbance of tillage practices based on the type of tillage equipment used, tillage depth, speed, and percent of soil surface distributed, and can range in value from zero to 200 with higher values indicating greater tillage intensity [31]. Data for these calculations come from Table F in Phase 2 of the ARMS survey. Conventional tillage is defined as a combination of tillage management practices used between harvest of the previous crop and harvest of current crop that result in a STIR value >80. Mulch till is defined as tillage management practices used between harvest of previous and current crop that have a STIR value ≤80, where soil is tilled but soil disturbance is low. No till is defined as the practice of not tilling between harvest of the cash crop.

After determining the tillage type used by each respondent (either conventional or conservation tillage) we calculated the share of respondents using these practices to evaluate their prevalence and trends between the two time periods for each crop, by ERS Resource Region (which depict the geographic specialization of U.S. commodity production [32] and by state for field corn and soybeans. As described in the ARMS documentation we applied weights to calculate these shares because of the probability-based sampling procedure used to collect ARMS responses [33].

**Herbicide usage by field corn (2016 and 2021) and soybeans (2012 and 2018) producers practicing conventional versus conservation tillage**

To compare herbicide usage by tillage type (i.e., conventional versus conservation tillage) we conducted mean comparison tests for three measures of herbicide usage: (1) percent of crop acres treated with the herbicide (PCT), (2) pounds of herbicide applied per treated acre (lbs./acre), and (3) the frequency of herbicide applications per acre (no. applications/acre) (note that this variable has historically been called “acre treatments”). Each measure was examined by application timing, i.e., total for the survey year; before/at planting (i.e., pre-emergence to the cash crop); and after planting (i.e., post-emergence to the cash crop). Table D of the ARMS Phase 2 questionnaire asks producers if any herbicides were used on the sample field during the crop year and if the answer is yes, producers are asked to list the herbicide name (using a list provided [33]), what form it takes (dry or liquid), whether the product was part of a tank mix (a mix of multiple herbicides or other pesticides), how much was applied, and the amount applied per application to the sample field. These responses are then matched to a list of each product’s active ingredient which is then used to estimate the amount (in pounds) of each chemical per product used. To calculate variables that were on a per acre basis we used the producers reported field size in acres.

Percent of crop acres treated with the herbicide is the maximum acres treated with any product containing the herbicide divided by the sample field size in acres. Pounds of herbicide applied per treated acre is calculated as the total pounds of the chemical applied to the sample field divided by the maximum treated acres times the number of applications of the product with the maximum value for treated acres; this value is representative of the single application rate (lbs ai/acre) for a given herbicide. The per acre herbicide application frequency (also known as acre treatments) is the maximum treated acres times the number of applications of the product with the maximum value for treated acres divided by the sample field size.

Pairwise mean comparisons were made separately for each crop (i.e., field corn and soybeans) and herbicide (i.e., 2,4-D, atrazine, and herbicide) to identify whether usage was statistically different for conventional versus conservation tillage and for pre- versus post-emergence application timings. Prior to mean comparison tests, we determined whether the data were parametric using the Shapiro-Wilk test. When parametric assumptions were met a t-test was used to determine if mean herbicide usage was statistically different between the two groups (i.e., conventional tillage versus conservation tillage). When parametric assumptions were not met a Wilcoxon signed rank sum or Kruskal-Wallis test was used to compare herbicide usage across tillage types. All tests were conducted using the ‘survey’ package (version 4.2.1) in R [34,35]. As with our calculation of shares for conservation practices, we conducted these tests with survey weights because of the probability-based sampling procedure used to collect ARMS responses [33].

**Classification and Regression Trees (CART) analysis predicting Conservation Tillage**

To understand the relationship between conservation tillage and herbicide usage we employ a Classification and Regression Trees (CART) analysis. CART is an analytical technique that splits a full dataset into more homogeneous groups through a binary recursive partitioning process. In doing this it helps researchers understand the most important explanatory variables in a dataset which can inform additional explanatory models or studies using causal inference techniques.

CART has been used extensively in epidemiology and medical settings since the mid-1980s to tease out which human behaviors or characteristics are most important for delivering medical interventions most effectively [36]. In these contexts, it is often the case that many human behaviors or characteristics confound each other, which makes use of standard statistical methods to determine which factors are most important to intervention delivery, such as multivariate linear regression, challenging and less useful. Further, practitioners have indicated that the results from regression models are harder for clinicians to interpret and apply in the clinical setting [37]. In the context of understanding drivers of conservation tillage adoption in agricultural production and the relative importance of herbicide usage, CART is a useful tool given that farmer behavior and production decisions are related and interactive, often confounded by multiple related factors.

CART is a simplified machine learning approach which can evaluate the relationship between many more variables relative to regression analysis, which means the cost of deciding which variables to analyze or include in a model is relatively low. Variables included in the analysis also do not need to follow a particular distribution, making it an advantageous alternative to regression analysis when data are not parametric. Further, there are limited ways in which the CART model can be adjusted or modified, which means its relatively easy to use. A tolerance threshold for the recursive partitioning process and an a priori minimum group size must be set, but otherwise model parameters or options are very limited. Similar to regression analysis, the key inputs to the CART analysis are an outcome variable and explanatory variables. Typically, researchers will test the model with a subset of observations from a dataset and then train it with a larger subset of the same dataset.

Results from the CART analysis are displayed in a tree composed of a hierarchical set of branches or nodes collectively indicating the level of importance of each explanatory variable in predicting the outcome variable of interest (in this case, adoption of conservation tillage). The first explanatory variable to split the data, which would create the first set of nodes, is considered the most predictive of the outcome variable. The second most predictive explanatory variable will create the second set of nodes in the tree, and so on. Each split of the data is meant to create more and more homogenous groups within the data. Tree depth (i.e., the number of hierarchical splits in the tree) can be restricted to ensure that final results are not overly complex to interpret, but this is a subjective decision left to the researcher. The value that the explanatory variable takes in predicting the value of the outcome variable is also displayed on the tree. CART trees could theoretically include splitting the data into so many groups that each observation (in our case observations are respondents in ARMS) is in its own terminal node, but this would not provide meaningful information. The researcher can adjust model parameters or “prune” the tree to ensure results are interpretable [38]. Pruning parameters include: minimum group size at terminal nodes, maximum tree depth (i.e., number of splits), cross validation error, complexity parameter (CP), and prediction accuracy.

The primary outcome of interest in the CART analysis is whether a producer is implementing conservation tillage versus conventional tillage. The primary explanatory variables of interest are the three indicating the level of herbicide usage, as described earlier in the methods section. The full list of variables used in the analysis can be found in S1 Appendix. We identified these variables by beginning with the full set of responses to the Phase 2 portion of the ARMS survey and selectively excluding survey questions that did have a known economic or agronomic relationship to conservation tillage or herbicide usage based on prior literature and/or expert feedback. The selection process was liberal, including any variable with an evidence-based or hypothetical relationship to herbicide use or usage. Through this process, we identified 67 variables to include in the soybean CART model and 90 for field corn. All included variables are described in S1 Appendix and each variable is identified based on the ARMS questionnaire survey question from which it was derived. Some minor data cleaning and recoding was necessary for some of these variables, but nothing was done that would change the interpretation of the survey questions as they are stated in the ARMS questionnaires.

CART analyses were run in R using the ‘rpart’ package (version 4.1.21) on the 2016 field corn responses (n=1,995) and the 2018 soybean responses (n=2,260) [39]. We chose to not run the model on the 2021 field corn responses given that the response rate in 2021 (n=968) drop significantly relative to 2016 and prior years of the ARMS field corn survey. It is not clear why the response rate in 2021 dropped so dramatically, although it may be attributable to survey distribution challenges that arose during the COVID-19 pandemic. This is an area to explore in future research.

We randomly selected 20% of the ARMS survey responses for each survey year for field corn and soybeans to test the model. We used the remaining 80% of the survey responses as the training data (and report results from the model runs on the training data in the results section). The minimum group size at the tree terminal nodes was 20, which is the default value in the ‘rpart’ package. We iteratively adjusted the maximum tree depth to maximize the prediction accuracy of our models. For field corn, maximum tree depth did not impact prediction accuracy so we left it unrestricted. For soybeans, the maximum tree depth was seven which provided the highest prediction accuracy relative to leaving the maximum depth unrestricted. We also evaluated the cross-validation error rate and the Complexity Parameter to ensure high model accuracy.

**Interpretation of results using ARMS responses**

ARMS questions are designed to ask producers about their production practices on a single field within their larger operation. Respondents are asked to list all of their fields, no matter how they define those fields individually, in the responses to the survey. Then ARMS enumerators pick a random field from each producer’s list of reported fields and instruct respondents to answer questions about production practices for the selected field only. Consequently, survey responses used for our analysis are for the random field selected by ARMS enumerators and do not represent the entirety of production decisions for each respondent. However, the ARMS survey sample population of producers is constructed so that it is nationally representative of the population of producers for a particular commodity. Since each field is randomly selected by ARMS enumerators we assume these fields too are nationally representative of the population of producers of a particular commodity, so we interpret our results accordingly.

**Results**

**Conservation tillage adoption by field corn and soybean producers**

In 2016, 41% of U.S. field corn producers were implementing conventional tillage on their fields and 59% were implementing conservation tillage, while in 2021, 28% were implementing conventional tillage and 72% were implementing conservation tillage. There was a 13-percentage point swing towards conservation tillage adoption amongst U.S. field corn producers between 2016 and 2021.

When parsed by ERS farm resource region [32], conservation tillage adoption in 2021 exceeded 90% in the Eastern Uplands and Prairie Gateway resource regions, which respectively saw 28 and 12 percentage point increases in adoption between 2016 and 2021 (Fig 1). In 2016, conservation tillage adoption was lowest in the Fruitful Rim resource region, but this region also saw the greatest increase in adoption (+33 points) between 2016 and 2021. All resource regions experienced an increase in conservation tillage adoption from 2016 to 2021 except for the Northern Great Plains. We do not have data for the Mississippi Portal region for field corn because the ARMS survey does not cover the states included in it (LA, MS, AR, and TN), even though a small amount of field corn production does occur there.

**Fig 1. For field corn, conservation tillage increased in all but one region between 2016 and 2021.** This chart shows the percentage of fields in the ARMS sample for field corn that reported using conservation tillage in 2016 (n=1,995) and 2021 (n=968). The only region that did not experience an increase in the rate of conservation tillage over this period was the Northern Great Plains. Data not reported for the Mississippi Portal because the ARMS field corn does not survey states in this region (including LA, MS, AR, and TN).

At the state level, conservation tillage adoption by field corn producers in 2021 exceeded 90% for Kansas (98%), Kentucky (97%), Illinois (91%) and Georgia (90%); conservation tillage adoption was lowest for Texas and Michigan (44%) field corn producers (Fig 2). Increases in conservation tillage adoption between 2016 and 2021 were highest for Ohio (+ 30 percentage points), Georgia (+ 28 points), and North Carolina and Texas (+ 24 points). Nebraska and New York saw very minor (- 0.5 points) declines in conservation tillage adoption, while conservation tillage adoption in Missouri fell by almost 8.5 points; there were concomitant and proportional increases in the utilization of conventional tillage in these states.

**Fig 2. For field corn, conservation tillage increased in all but three states from 2016 to 2021.** This chart shows the percent of fields that reported using conservation tillage in 2016 (n=1,995) and 2021 (n=968) by states included in the ARMS survey. The ARMS survey is only administered to states representing the vast majority of U.S. field corn production and so not all states are represented in these results.

In 2012, 25% of U.S. soybean producers were implementing conventional tillage and 75% were implementing conservation tillage, and this remained largely unchanged in 2018 with 22% implementing conventional tillage and 78% implementing conservation tillage. Nationally, there was only a 3-percentage point swing towards conservation tillage adoption amongst U.S. soybean producers between 2012 and 2018.

When parsed by ERS farm resource region, conservation tillage adoption in 2018 exceeded 90% in the Eastern Uplands, Southern Seaboard, and Prairie Gateway resource regions (Fig 3), though the latter two regions saw minor declines (- 2-5 percentage points) between 2012 and 2018, while in the Eastern Uplands there was only a minor increase in adoption of 7 points. Conservation tillage adoption was lowest in the Mississippi Portal and Northern Crescent regions, which both saw minor increases (+ 2-4 points) between 2012 and 2018. The Northern Great Plains region saw the greatest increase in adoption (i.e., + 17 points), resulting in overall conservation tillage adoption by almost two thirds of soybean producers in the region by 2018.

**Fig 3. For soybeans, conservation tillage increased in all but two regions from 2012 to 2018.** This chart shows the percentage of fields in the ARMS sample for soybeans that reported using conservation tillage in 2012 (n=1,791) and 2018 (n=2,260) by ERS Resource Region. No data are reported for the Fruitful Rim or Range and Basin regions because the ARMS soybean survey does not cover states in these regions even though some soybean production, albeit a small amount, does occur in them.

At the state level, conservation tillage adoption by soybean producers in 2018 exceeded 90% for Kentucky (100%), Tennessee (100%), Virginia (98%), Nebraska (96%), North Carolina and Missouri (91%); conservation tillage adoption was lowest for Arkansas (29%), Minnesota (37%) and Louisiana (43%) soybean producers (Fig 4). Increases in conservation tillage adoption between 2012 and 2018 were highest for North Dakota (+ 20 percentage points), Missouri (+ 12 points), and Tennessee (+ 11 points). Conservation tillage adoption by soybean producers declined in multiple states between 2012 and 2018, i.e., in Kansas (- 9 points), North Carolina and Ohio (- 7 points), Indiana (- 4 points) and Arkansas (- 1 points); there were concomitant and proportional increases in the utilization of conventional tillage in these states.

**Fig 4. For soybeans, in most states conservation tillage adoption increased from 2012 to 2018.** This chart shows the percentage of fields in the ARMS survey for soybeans that reported using conservation tillage in 2012 (n=1,791) and 2018 (n=2,260) among states included in the ARMS survey. The ARMS survey is only administered to states representing the vast majority of U.S. soybean production and so not all states are represented in these results.

**Herbicide usage by field corn and soybean producers practicing conventional versus conservation tillage**

Overall, for both field corn and soybeans, glyphosate usage (measured as percent crop acres treated) was higher than that of atrazine (for field corn) or 2,4-D (for soybeans). In the surveyed years, on average 77% of field corn acres received glyphosate treatments compared with 62 and 65% of field corn acres receiving atrazine treatments in 2016 and 2021, respectively. For soybean acres on average,82-95% received glyphosate treatments compared to 15-19% of acres receiving 2,4-D treatments.

Average per acre single application rates for glyphosate were 0.91-1.00 lbs. active ingredient (ai)/acre for both field corn and soy, but field corn acres tended to receive only one application per year on average while soybean acres received between 1.30 and 1.60 applications per year, indicating that individual application rates for glyphosate in soybeans tended to be lower than those in field corn (Table 1).

**Table 1. Average Usage of 2,4-D, Atrazine, and Glyphosate among U.S. Corn (2016 and 2021) and Soybean (2012 and 2018) Producers.**

| **Crop** | **Herbicide** | **Year** | **Percent Crop Treated (%)** | **Average Single Application Rate (lbs ai/acre)** | **Annual Number of Applications per Acre** |
| --- | --- | --- | --- | --- | --- |
| Corn | Atrazine | 2016 (n=1,995) | 61.8 | 0.93 | 0.75 |
|  |  | 2021 (n=968) | 64.7 | 0.88 | 0.84 |
|  | Glyphosate | 2016 (n=1,995) | 77.4 | 0.93 | 1.02 |
|  |  | 2021 (n=968) | 76.5 | 1.00 | 1.00 |
| Soy | Glyphosate | 2012 (n=1,791) | 95.0 | 0.96 | 1.61 |
|  |  | 2018 (n=2,260) | 81.4 | 0.91 | 1.30 |
|  | 2,4-D | 2012 (n=1,791) | 15.0 | 0.54 | 0.15 |
|  |  | 2018 (n=2,260) | 19.2 | 0.52 | 0.22 |

The average percent of field corn acres treated with either atrazine or glyphosate under conservation tillage was 7 and 10 percentage points higher (p ≤ 0.01) in 2016 relative to acres treated under conventional tillage. In 2021, there were no statistically significant differences in the percentage of acres treated with atrazine or glyphosate by tillage type among corn acres (S1 Table). There were no consistent differences in single application rates for atrazine or glyphosate by tillage type in 2016, though in 2021 glyphosate single application rates on corn acres under conventional tillage were 0.13 lbs ai/acre lower (p ≤ 0.01) than those under conservation tillage; application rates on conventional tillage acres fell relative to 2016 while rates on conservation tillage acres remained steady at 0.90-0.95 lbs. ai/acre. On conservational tillage acres, generally more than one application of glyphosate was made per acre in 2016 and 2021, significantly (p ≤ 0.001 for 2016) more frequent than on conventional tillage acres, which received on average 0.83-0.89 applications per acre. Similarly, conservation tillage acres received more (p ≤ 0.01) frequent applications (0.79-0.89) of atrazine than conventional tillage acres (0.65-0.73).

The same general trends were also generally observed with soybean acres (S2 Table), with the percent of acres treated with either 2,4-D or glyphosate, and under conservation tillage, 15-16 and 7 percentage points higher (p ≤ 0.01) for 2,4-D or glyphosate, respectively. There were no consistent statistically significant differences in single application rates for 2,4-D or glyphosate by tillage type in 2012 or 2018. On conservational tillage acres, glyphosate was applied 0.16-0.26 times more frequently than on conventional tillage acres, while 2,4-D applications were 0.17 times more frequent on conservation tillage acres (p ≤ 0.001).

The apparent higher level of herbicide usage for both corn and soybean acres under conservation tillage (relative to acres using conventional tillage) is specifically for applications made pre-emergent to the crop (Fig 5, S1 and S2 Tables). In corn, while the percent of crop treated post-emergence was generally not different between conservation and conventional tillage, the percent of conservation tillage crop treated pre-emergence was 10-17 percentage points and 21-22 percentage points higher (p ≤ 0.001) for atrazine and glyphosate, respectively. Likewise, the frequency of pre-emergence applications on conservation tillage acres was 0.24-0.25 and 0.13-0.19 times greater (p ≤ 0.001) for atrazine and glyphosate, respectively. In soy, while the percent of crop treated post-emergence was unchanged (or in the case of 2012 when conservation tillage acres treated with glyphosate fell by 5 percentage points (p ≤ 0.01), the percent of conservation tillage acres treated pre-emergence was 13-15 percentage points and 29 percentage points higher (p ≤ 0.001) for 2,4-D and glyphosate, respectively. Likewise, the frequency of pre-emergence applications on conservation tillage acres increased by 0.15-0.17 and 0.32-0.34 times (p ≤ 0.001) for 2,4-D and glyphosate, respectively.

**Fig 5. Pre-emergent percent crop treated is significantly higher for conservation tillage (field corn and soybeans).** This chart shows average percent crop treated across field corn and soybean fields (2016, n=1,995 and 2021, n=968) and soybean (2012, n=1791and 2018, n=2,260) for Glyphosate, Atrazine and 2,4-D in the pre-emergent and post-emergent crop production phases.

**CART analysis results**

**Predictors of Conservation Tillage Among Field Corn and Soybean Producers**

The results of our CART analysis predicting conservation tillage usage for field corn (using the 2016 ARMS responses) are presented in Fig 6; explanatory variables are described in detail in S1 Appendix. The prediction accuracy of this model is 68.5%, meaning that if the model was applied to other data using the same explanatory variables it would accurately predict a field or farms use of conservation tillage 68.5% of the time. While a higher level of prediction accuracy is always preferred, we do not have other CART models of conservation tillage to compare prediction accuracy values, so it is unclear how we could adjust our model to improve it. This is an area that warrants future research.

The pre-emergent percent of crop acres treated with glyphosate most strongly predicts conservation tillage adoption, with the data splitting at 18% of acres treated (node A in Fig 6). This means that if ≥18% of a sample field’s acres were treated with glyphosate the model predicts that the field will be in conservation tillage with a predicted probability of 0.86 (node B). Twenty-two percent of the sample is categorized in this group and the remaining 78% of the sample require a more complex set of variables to explain conservation tillage adoption (beginning at node C). If the pre-emergent percent of crop acres treated with glyphosate is <18% than the model still predicts that a producer will use conservation tillage but at a lower rate (0.58 predicted probability).

Conditional on treating less than 18% of the field acres with glyphosate, the next most predictive explanatory variable was whether the producer’s fields used a crop rotation, followed by whether the farmland was classified as highly erodible land (HEL) definition based on USDA Natural Resource Conservation Service standards (see S1 Appendix for full description). If crop rotations were used (i.e., if a crop other than corn was grown in a given field the previous year) the model’s predicted probability of conservation tillage adoption rose from 0.58 to 0.64 and the model predicts that the field would be in conservation tillage (node D). Further, if a field was in a crop rotation and also classified as HEL then the predicted probability of conservation tillage adoption rises to 0.86 and the model predicts the field will be in conservation tillage (node E). Farm size and field acres planted play a minor mediating role in predicting conservation tillage.

The largest final group size included producers with a farm size ≥48 acres, on land not classified as HEL, using crop rotations and those who treated less than 18% of their acres with glyphosate in the pre-emergent phase of production (node F). For these producers, conservation tillage is the predicted tillage type and the predicted probability is 0.63. For smaller farms and fields conventional tillage is predicted, but this group represents only 3% of the training data sample.

**Fig 6. For field corn, pre-emergent glyphosate percent crop treated was the strongest predictor of conservation tillage.** This chart shows CART analysis results for 2016 field corn responses (n=1,995). Blue shading indicates that the model predicts the sub-group is using conservation tillage. The darkness of the blue indicates the magnitude of the predicted probability, with a darker color indicating a higher predicted probability. Green shading indicates that the model predicts conventional tillage use. The word “conservation” or “conventional” in each blue or green box indicates which tillage type the model predicts for that subgroup of producers. The middle number that is expressed as a decimal indicates the probability of conventional tillage adoption. For boxes that are shaded in blue where conservation tillage is predicted the predicted probability of conservation tillage is calculated as 1 minus the value in the box (i.e., at node B, the predicted probability of conservation tillage is 1-0.14=0.86). The last number in the boxes that are expressed as percentages indicate the share of the training sample that is categorized into that subgroup (i.e. at node B, the model categorizes 22% of respondents into this subgroup).

Fig 7 presents results from the CART analysis of 2018 soybean producer responses. The prediction accuracy of this model is 72.2%, higher than the field corn model. Similar to the results for field corn, the most predictive explanatory variable for conservation tillage is the pre-emergent percent of field acres treated with glyphosate (node A). If the value is > 56% the model predicts that the field will be in conservation tillage adoption (predicted probability of 0.90). The model predicted that 41% of the fields are categorized into this terminal node, the second largest group size of any others in the terminal nodes. If the percent of acres treated with glyphosate in the pre-emergent phase is less than 56% then conservation tillage use drivers differ by ERS resource region. For producers in the Eastern Uplands, Heartland, Northern Great Plains, Prairie Gateway and Southern Seaboard resource regions, conservation tillage use is predicted (probability = 0.72) when producers apply less than 1.4 lbs glyphosate per acre per application (node B).

For producers who treat less than 56% of acres with glyphosate pre-emergent and are in the Northern Cresent or Mississippi Portal resource regions, conservation tillage is predicted if a field that is less than 4.9 acres (predicted probability = 0.83). However, this group represents only 2% of the sample population.

**Fig 7. For soybeans, pre-emergent glyphosate percent crop treated was also the strongest predictor of conservation tillage.** This chart shows CART analysis results for 2018 soybean responses (n=2,260). Blue shading indicates that the model predicts the sub-group is using conservation tillage. The darkness of the blue indicates the magnitude of the predicted probability, with a darker color indicating a higher predicted probability value. Green shading indicates that the model predicts conventional tillage use. The word “conservation” or “conventional” in each blue or green box indicates which tillage type the model predicts for that subgroup of producers. The middle number that is expressed as a decimal indicates the probability of conventional tillage adoption. For boxes that are shaded in blue where conservation tillage is predicted the predicted probability of conservation tillage is calculated as 1 minus the value in the box (i.e., at node B, the predicted probability of conservation tillage is 1-0.14=0.86). The last number in the boxes that are expressed as percentages indicate the share of the training sample that is categorized into that subgroup (i.e. at node B, the model categorizes 22% of respondents into this subgroup).

**Discussion**

Our analyses provide the latest information on conservation tillage adoption among U.S. corn and soybean producers and a novel analysis of the relative importance of commonly used herbicides for its adoption. In theory, the 2022 Census of Agriculture would provide the most up to date information on tillage practices. It was released in February 2024 and contains information on adoption of conservation tillage among corn and soybean producers. However, this commodity specific data on conservation tillage adoption can only be accessed through the restricted use Census of Agriculture microdata or by requesting a special tabulation from the National Agricultural Statistics Service; the authors were unable to acquire these data prior to publication to make comparisons to our results. Further, the Census of Agriculture asks producers whether they are adopting conservation, no-till, or conventional tillage and does not specifically calculate a STIR rating as ARMS does. These self-reported measures of tillage used in the Census of Agriculture are subject to more uncertainty because there is such a wide variety of tillage practices within each type. STIR is a more objective measure that is preferable to use where possible. Consequently, while there are more updated data available, our results do represent the latest and most accurate information on conservation tillage adoption among U.S. field corn and soybean producers that currently exists in the literature. Prior to our study, the latest information describing nationally representative prevalence and trends in conservation tillage adoption are from 2018, nearly 6 years ago, that included data for field corn through 2016 and for soybeans through 2012 at the national, state and region levels [9]. The updated national, regional, and state level prevalence levels and trends in conservation tillage adoption provide essential information that policymakers can use to continue to track conservation tillage adoption in U.S. commodity production.

What we find, as of the latest sampled years (i.e., 2018 for soybean and 2021 for field corn), is that roughly three quarters of U.S. acres of these crops were using conservation tillage (either mulch tillage or no till). While there was a 13% increase in field corn acres under conservation tillage between 2016 and 2021, soybean acres under conservation tillage remained steady between 2012 and 2018. The trends observed here are generally consistent with the trends between 2010 and 2016 for field corn and 2006 to 2012 for soybeans. Overall, this indicates a steady upward trajectory in adoption of conservation tillage nationally over the last 20 years. While most regions and states experienced increases in conservation tillage, there were some states that experienced declines. For field corn, Missouri experienced an 11% decline, while the trend for conservation tillage was nearly flat for Nebraska, New York, and South Dakota. For soybeans, Indiana, Kansas, North Carolina and Ohio had between 4% and 9% declines in adoption. One recent study discussed the importance of tracking more localized trends in the adoption and dis-adoption of conservation tillage because a shift to conventional tillage has important implications for soil carbon sequestration (which requires longer term use of conservation tillage) and the stability of voluntary carbon markets and participation among U.S. farmers [40]. Our results support the idea of tracking conservation tillage not just nationally but at more granular geographic scales.

The information provided here is particularly important in the context of the recent infusion of funds into agricultural conservation programs in the Inflation Reduction Act intended to increase assistance to producers for adopting practices such as conservation tillage and no-till [41]. In addition, our analysis of conservation tillage adoption in the context of herbicide usage is especially opportune given label mitigation changes for 2,4-D, atrazine and glyphosate being considered by EPA both as part of routine registration review and EPA’s accelerated efforts to meet its consultation obligations under the Endangered Species Act [42,43]. To meet its pesticide registration obligations under the Federal Insecticide, Rodenticide and Fungicide Act (FIFRA), EPA is considering changes to allowable uses on these herbicide labels that could directly constrain producers’ use of herbicides to facilitate conservation tillage, for example, by discouraging the pre-emergence use of atrazine [30]. Under the ESA, EPA is required by law to consult with the U.S. Fish and Wildlife Service (FWS) and the National Marine Fisheries Services (NMFS) to ensure that pesticide registrations do not imperil threatened and endangered species or their critical habitats [44]. One of the approaches EPA has proposed to mitigate off-field risks to threatened and endangered species is by requiring producers to utilize on- and off-field practices that mitigate runoff and soil erosion, which includes conservation tillage [42]. Our data on the prevalence of conservation tillage amongst US field corn and soybean illustrates both the potential magnitude of impact of these changes, and the complexity of the drivers of even just conservation tillage practices.

Prior studies looking at the relationship between herbicide usage and conservation tillage adoption used fairly coarse measures of average herbicide usage, and most of these studies are from over two decades ago, largely before the commercialization of corn and soybean seed varieties genetically-engineered to be resistant to glyphosate [45,46]. Our updated analysis allows us to better account for the potential impact of the commercialization of herbicide-resistant field corn and soybean varieties on the relationship between herbicide usage and conservation tillage adoption. ARMS data allows us to provide a more nuanced picture of the differences in herbicide application between conventional and conservation tillage because it asks about application timing (i.e., pre- versus post-emergent) and also asks producers about a range of agronomic and economic factors in addition to asking about their herbicide usage and tillage practices.

Higher percent crop acres treated for field corn and soybean producers practicing conservation tillage relative to those practicing conventional tillage implies overall greater usage of 2,4-D, atrazine and glyphosate pre-emergence for corn and soybean producers practicing conservation tillage. Specifically, our analyses appear to suggest that producers practicing conservation tillage on their fields are shifting the timing of their applications of these herbicides to the pre-emergence timing window. We don’t find that single application rates for these herbicides significantly differ between fields under conservation and conventional tillage, but the percent of crop acres treated pre-emergence and the frequency of pre-emergence applications is higher in fields under conservation tillage compared with fields under conventional tillage. Note that the calculation of the application frequency parameter accounts for all corn and soybean field acres, respectively, so there is a potential for downward bias for an herbicide like 2,4-D that is typically used on a relatively small percentage of overall planted soybean acres. These results were indicated by both our simple mean comparison tests of herbicide usage indicators and the CART analysis. The latter further suggested that for field corn producers, fields that had conservation tillage were more likely to have implemented a crop rotation and more likely to have a field classified as highly erodible. These results contrast with the work done on this topic over two decades ago, many of which found minimal differences in herbicide usage between producers using conservation tillage and those using conventional tillage [23–25].

Our results highlight the complicated nature of weed management in many agricultural systems, and the inherent trade-offs therein. Weeds pose a major threat to productive agricultural systems, and effective direct weed management tools available to producers are arguably limited to herbicides, tillage, and cropping practices (e.g., rotations) [47]. Removing any one of these three tools increases reliance on the others, and so producers practicing conservation tillage (especially no-till) largely depend on herbicides for weed control [4,47,48]. As herbicide-resistant field corn and soybean seed varieties have become commonplace producers have become accustomed to being able to use largely or entirely broad-spectrum herbicides such as 2,4-D, dicamba or glyphosate for post-emergence weed control [7,48,49]. An axiom of modern farming in the US is the importance of seeding into a field as close to entirely free of weeds as possible, and pre-plant weed control is achieved either through tillage, or in the case of producers using conservation tillage, with herbicides [48]. Therefore, our results – i.e., that producers practicing conservation tillage would appear to treat a higher percent of acreage with herbicides pre-emergence than those practicing conventional tillage – seem to intuitively represent the replacement of tillage by chemical weed control for the preparation of a weed-free seed bed.

Our results specifically point to the important role that crop rotations can have in aiding in weed management by allowing producers to rotate herbicides and cultural weed management practices (e.g., row spacing) alongside crops, and by reducing the chances that single weed species become unmanageable [5,7]. There is growing evidence that the use of crop rotations or other cultural weed management practices such as cover cropping, in addition to conservation tillage, further optimizes weed management programs and allow producers to reduce herbicide usage [4,5,48].

Our CART analysis provides a more comprehensive picture of the relationship between herbicide usage and conservation practice adoption in the context of other production practices, going beyond prior studies that simply compare mean usage of herbicides across different tillage types. This analysis helps us to not only understand how herbicide usage differs between conventional and conservation tillage, it also allows us to rank the importance of herbicide usage in the context of many other production practices, farm size, and the geographic region and state of production. Our results indicate that the percent of crop treated with glyphosate is central to the adoption of conservation tillage for both field corn and soybeans. However, other factors such as whether the field is in certain production regions or whether the field is considered highly erodible are also key factors that predict conservation tillage. These findings are intuitive because conservation tillage helps to preserve soil health, and if fields are classified as highly erodible a producer is actually required to have a conservation plan that includes the use of reduced tillage practices [50,51]. Further, soils vary geographically, so we would expect to see variation in conservation tillage adoption across regions. Further research is needed to tease out why some regions have higher or lower adoption levels. Additional studies are also need to evaluate why larger farms, conditional on other factors (as illustrated in Fig 6), would be more likely to adoption conservation tillage.

Results from this study also demonstrate the potential for using CART to better understand decision making in agricultural production. There are limited examples of CART models being applied in the agricultural setting; one example is a recent working paper [52] that evaluates the relationship between conservation practice adoption and crop insurance use. CART models could, however, become a more common modeling tool used because these models can be applied to better understand farmer decision making when many factors confound one another. CART models also have advantages over multiple linear regression in that data used in CART analyses require fewer assumptions (i.e., data do not need to meet parametric assumptions) and variables that would typically be considered collinear can all be included in CART models. Moving forward, CART models could be used to explore how farm characteristics, production practices or other agronomic or economics factors are related to adoption of cover crops or other conservation practices that have environmental or climate benefits. CART models are also used in preparation for causal inference studies because model results can help identify variables that confound the causal relationship between key explanatory and outcome variables. Few previous studies have evaluated the causal relationship between policy changes restricting or otherwise impacting herbicide usage and conservation tillage or other production practices, and the results of our initial CART models could help inform such future studies.

**Conclusions**

Results from this study provide further evidence that conservation tillage adoption amongst U.S. field corn and soybean producers has continued to increase over time, with roughly 75% of these producers practicing either mulch tillage or no-till as of 2018 (soybean) and 2021 (field corn). With this said, attention should be drawn to regional and state variability in adoption that points to the potential for dynamic trends in producers switching back to conventional tillage. Our analysis of ARMS data indicates that producers using conservation tillage practices apply relatively more herbicides – atrazine and glyphosate for field corn, and 2,4-D and glyphosate for soybeans – prior to crop planting than producers using conventional tillage, but the overall use (in terms of pounds) is not significantly different between conservation and conventional tillage. CART models confirmed that pre-emergent use of 2,4-D, atrazine, or glyphosate was the most important predictor variable for field corn or soybean adoption of conservation tillage. Therefore, any label changes that would directly impact the ability of producers to apply these herbicides pre-emergent to the crop could impair their continued practice of conservation tillage. Additionally, CART model results pointed to the importance of crop rotations and other cultural weed management practices in facilitating the practice of conservation tillage, which highlights the role that Integrated Weed Management practices more generally have in optimizing weed control so as to support conservation agricultural practices.

**Acknowledgements**

The authors would like to thank reviewers at the US Department of Agriculture Economic Research Service and Office of the Chief Economist for their feedback and suggestions that greatly improved the condition of this research article.

**References**

1.	Carlson GA. Costs Impacts if Atrazine or Triazines Were Not Available to Growers. Triazine Herbicides: Risk Assessment. American Chemical Society; 1998. pp. 35–48. doi:10.1021/bk-1998-0683.ch004

2.	Mitchell PD. Market-level assessment of the economic benefits of atrazine in the United States. Pest Manag Sci. 2014;70: 1684–1696. doi:10.1002/ps.3703

3.	Duke SO. The history and current status of glyphosate. Pest Management Science. 2018;74: 1027–1034. doi:10.1002/ps.4652

4.	Moyer J, Roman E, Lindwall C, Blackshaw R. Weed management in conservation tillage systems for wheat production in North and South America. Crop Protection. 1994;13: 243–259.

5.	Nichols V, Verhulst N, Cox R, Govaerts B. Weed dynamics and conservation agriculture principles: a review. Field Crops Research. 2015;183: 56–68.

6.	Chauhan B, Singh G, Mahajan G. Ecology and management of weeds under conservation agriculture: a review. Crop Protection. 2012;38: 57–65.

7.	Locke M, Reddy K, Zablotowicz R. Weed management in conservation crop production systems. Weed Biology and Management. 2002;2: 123–132.

8.	Shrestha A, Lanini T, Wright S, Vargas R, Mitchell J. Conservation Tillage and Weed Management. Davis, CA: University of California, Division of Agriculture and Natural Resources; 2006. Report No.: 8200.

9.	Claassen R, Bowman M, McFadden J, Smith D, Wallander S. Tillage intensity and conservation cropping in the United States. Washington DC: USDA Economic Research Service; 2018.

10.	Wade T, Claassen R. Modeling no-till adoption by corn and soybean producers: Insights into sustained adoption. Journal of Agricultural and Applied Economics. 2017;49: 186–210.

11.	Fernandez-Cornejo J, Nehring RF, Osteen C, Wechsler S, Martin A, Vialou A. Pesticide use in US agriculture: 21 selected crops, 1960-2008. USDA-ERS Economic Information Bulletin. 2014.

12.	Fernandez-Cornejo J, Hallahan C, Nehring RF, Wechsler S, Grube A. Conservation tillage, herbicide use, and genetically engineered crops in the United States : the case of soybeans. 2012 [cited 8 Jun 2023]. Available: https://mospace.umsystem.edu/xmlui/handle/10355/35118

13.	Krause MA, Black JR. Optimal Adoption Strategies for No-till Technology in Michigan. Review of Agricultural Economics. 1995;17: 299–310. doi:10.2307/1349575

14.	Uri ND. Factors affecting the use of conservation tillage in the United States. Water, Air, and Soil Pollution. 1999;116: 621–638.

15.	Lal R, Kimble JM. Conservation tillage for carbon sequestration. Nutrient Cycling in Agroecosystems. 1997;49: 243–253. doi:10.1023/A:1009794514742

16.	Kaye JP, Quemada M. Using cover crops to mitigate and adapt to climate change. A review. Agron Sustain Dev. 2017;37: 4. doi:10.1007/s13593-016-0410-x

17.	Gebhardt MR, Daniel TC, Schweizer EE, Allmaras RR. Conservation tillage. Science. 1985;230: 625–630.

18.	Perry ED, Moschini G, Hennessy DA. Testing for Complementarity: Glyphosate Tolerant Soybeans and Conservation Tillage. American Journal of Agricultural Economics. 2016;98: 765–784. doi:10.1093/ajae/aaw001

19.	Lin B-H, Taylor H, Delvo H, Bull L. Factors Influencing Herbicide Use in Corn Production in the North Central Region. Review of Agricultural Economics. 1995;17: 159–169. doi:10.2307/1349729

20.	Benbrook CM. Trends in glyphosate herbicide use in the United States and globally. Environ Sci Eur. 2016;28: 3. doi:10.1186/s12302-016-0070-0

21.	Economic Research Service, Natural Resources Conservation Service. The Economic and Environmental Benefits and Costs of Conservation Tillage. Washington, DC: US Department of Agriculture; 1998 Feb.

22.	Economic Research Service. The economic and environmental costs of conservation tillage. Washington DC: USDA; 1998. Available: https://archive.org/details/economicsenviron00unit

23.	Duffy M, Hanthorn M. Returns to corn and soybean tillage practices. US Department of Agriculture, Economic Research Service; 1984.

24.	Logan TJ, Davidson JL, Baker, Overcash MR. Effects conservation tillage on ground water quality: nitrates and pesticides. Chelsea, MI: Lewis Publishers, Inc.; 1987.

25.	Bull L, Delvo H, Sandretto C, Lindamood B. Analysis of Pesticide Use by Tillage System in 1990, 1992, and 1992 Corn and Soybeans. Washington, D.C.: USDA Economic Research Service; 1993 Oct. Report No.: 32.

26.	Kellogg RL, Nehring RF, Grube A, Goss DW, Plotkin S. Environmental indicators of pesticide leaching and runoff from farm fields. Agricultural productivity: Measurement and sources of growth. Springer; 2002. pp. 213–256.

27.	Perry ED, Ciliberto F, Hennessy DA, Moschini G. Genetically engineered crops and pesticide use in U.S. maize and soybeans. Science Advances. 2016;2: e1600850. doi:10.1126/sciadv.1600850

28.	Environmental Defense Fund. Conservation’s Impact on the Farm Bottom Line. Environmental Defense Fund, Soil Health Partnership, K-COE ISOM; 2021. Available: https://business.edf.org/wp-content/blogs.dir/90/files/Conservation-Impact-On-Farm-Bottom-Line-2021.pdf

29.	Maggie Monast, Laura Sands, Alan Grafton. Farm Finance and Conservation: How stewardship generates value for farmers, lenders, insurers and landowners. Environmental Defense Fund; 2018 Sep. Available: https://www.edf.org/sites/default/files/documents/farm-finance-report.pdf

30.	US Environmental Protection Agency. Proposed Revisions to the Atrazine Interim Registration Review Decision, Case Number 0062. 2022 [cited 28 Feb 2024]. Available: https://www.regulations.gov/document/EPA-HQ-OPP-2013-0266-1625

31.	Natural Resource Conservation Service. Soil Tillage Intensity Rating methodology. Washington, DC: US Department of Agriculture; 2008. Available: https://www.nrcs.usda.gov/sites/default/files/2022-11/CEAP-Croplands-2008-Methodology-SoilTillageIntensityRating.pdf

32.	Economic Research Service. ERS Farm Resource Regions. Washington, DC: US Department of Agriculture; 2000. Available: https://www.ers.usda.gov/publications/pub-details/?pubid=42299

33.	Economic Research Service. Agricultural Resource Management Survey documentation. Washington, DC: US Department of Agriculture; 2023 Dec. Available: https://www.ers.usda.gov/data-products/arms-farm-financial-and-crop-production-practices/documentation/

34.	Lumley T. Analysis of Complex Survey Samples. 2023. Available: https://cran.r-project.org/web/packages/survey/survey.pdf

35.	R Core Team. R: A language and environment for statistical computing. Austria; 2023. Available: https://www.R-project.org

36.	Lemon SC, Roy J, Clark MA, Friedmann PD, Rakowski W. Classification and regression tree analysis in public health: Methodological review and comparison with logistic regression. ann behav med. 2003;26: 172–181. doi:10.1207/S15324796ABM2603_02

37.	Lewis RJ. An Introduction to Classification and Regression Tree (CART) analysis. Annual Meeting of the Society for Academic Emergency Medicine; 2000; San Francisco, CA. Available: https://citeseerx.ist.psu.edu/document?repid=rep1&type=pdf&doi=6d4a347b99d056b7b1f28218728f1b73e64cbbac

38.	Causal Analysis/Diagnosis Decision Information System. Classification and Regression Tree Analysis overview. Washington, DC: Environmental Protection Agency; 2024 Jan. Available: https://www.epa.gov/caddis-vol4/basic-analyses-1#assumptions

39.	Thernaeu T, Atkinson B, Ripley B. Recursive partitioning for classification, regression, survival trees. 2023. Available: https://cran.r-project.org/web/packages/rpart/rpart.pdf

40.	Sawadgo W, Plastina A. The Invisible Elephant: Disadoption of Conservation Practices in the United States. Choices Magazine. 2022 [cited 6 Jun 2023]. Available: https://www.choicesmagazine.org/choices-magazine/submitted-articles/the-invisible-elephant-disadoption-of-conservation-practices-in-the-united-states

41.	US Department of Agriculture. Inflation Reduction Act Investment in USDA’s FPAC Programs. In: Farmers.gov [Internet]. 12 Sep 2022 [cited 26 Feb 2024]. Available: https://www.farmers.gov/loans/inflation-reduction-investments

42.	U.S. Environmental Protection Agency. EPA Releases Draft Strategy to Better Protect Endangered Species from Herbicide Use | US EPA. Jul 2023 [cited 26 Feb 2024]. Available: https://www.epa.gov/pesticides/epa-releases-draft-strategy-better-protect-endangered-species-herbicide-use

43.	U.S. Environmental Protection Agency. EPA Publishes Update on Vulnerable Species Pilot. Washington, DC; 2023 Nov. Available: https://www.epa.gov/pesticides/epa-publishes-update-vulnerable-species-pilot

44.	US Environmental Protection Agency. About the Endangered Species Protection Program. Feb 2024 [cited 28 Feb 2024]. Available: https://www.epa.gov/endangered-species/about-endangered-species-protection-program

45.	Economic Research Service. Agricultural Resources: Inputs. Washington, DC: US Department of Agriculture; 1993 Oct p. 41.

46.	Fuglie KO. Conservation Tillage and Pesticide Use in the Cornbelt. Journal of Agricultural and Applied Economics. 1999;31: 133–147. doi:10.1017/S0081305200028831

47.	Derksen DA, Blackshaw RE, Boyetchko SM. Sustainability, conservation tillage and weeds in Canada. Canadian Journal of Plant Science. 1996;76: 651–659.

48.	Price AJ, Balkcom KS, Culpepper SA, Kelton JA, Nichols RL, Schomberg H. Glyphosate-resistant Palmer amaranth: a threat to conservation tillage. Journal of Soil and Water Conservation. 2011;66: 265–275.

49.	Kniss AR. Genetically Engineered Herbicide-Resistant Crops and Herbicide-Resistant Weed Evolution in the United States. Weed Science. 2018;66: 260–273. doi:10.1017/wsc.2017.70

50.	Food Security Act. 1985. Available: https://www.congress.gov/bill/99th-congress/house-bill/2100

51.	Langdale GW, West LT, Bruce RR, Miller WP, Thomas AW. Restoration of eroded soil with conservation tillage. Soil technology. 1992;5: 81–90.

52.	Ifft J, Jodlowski M. Farm Heterogeneity and Leveraging Federal Crop Insurance for Conservation Practice Adoption. 2023. Available: https://ageconsearch.umn.edu/record/329679/
